# IVF for premature ovarian failure: first reported births using oocytes donated from a twin sister

**DOI:** 10.1186/1477-7827-8-31

**Published:** 2010-03-25

**Authors:** Eric Scott Sills, Adam C Brady, Ahmed B Omar, David J Walsh, Umme Salma, Anthony PH Walsh

**Affiliations:** 1 Division of Reproductive Endocrinology, Sims IVF, Dublin, Ireland; 2 Department of Obstetrics and Gynaecology, School of Medicine, Royal College of Surgeons in Ireland, Dublin, Ireland; 3 The Sims Institute, Rosemount Hall, Dundrum Road, Dundrum, Dublin 14, Ireland

## Abstract

**Background:**

Premature ovarian failure (POF) remains a clinically challenging entity because in vitro fertilisation (IVF) with donor oocytes is currently the only treatment known to be effective.

**Methods:**

A 33 year-old nulligravid patient with a normal karyotype was diagnosed with POF; she had a history of failed fertility treatments and had an elevated serum FSH (42 mIU/ml). Oocytes donated by her dizygotic twin sister were used for IVF. The donor had already completed a successful pregnancy herself and subsequently produced a total of 10 oocytes after a combined FSH/LH superovulation regime. These eggs were fertilised with sperm from the recipient's husband via intracytoplasmic injection and two fresh embryos were transferred to the recipient on day three.

**Results:**

A healthy twin pregnancy resulted from IVF; two boys were delivered by caesarean section at 39 weeks' gestation. Additionally, four embryos were cryopreserved for the recipient's future use. The sister-donor achieved another natural pregnancy six months after oocyte retrieval, resulting in a healthy singleton delivery.

**Conclusion:**

POF is believed to affect approximately 1% of reproductive age females, and POF patients with a sister who can be an oocyte donor for IVF are rare. Most such IVF patients will conceive from treatment using oocytes from an anonymous oocyte donor. This is the first report of births following sister-donor oocyte IVF in Ireland. Indeed, while sister-donor IVF has been successfully undertaken by IVF units elsewhere, this is the only known case where oocyte donation involved twin sisters. As with all types of donor gamete therapy, pre-treatment counselling is important in the circumstance of sister oocyte donation.

## Background

Patient age and number of prior failed fertility treatments have trended upward in recent years, and both factors have an adverse impact on pregnancy rates with IVF. Among women presenting for fertility treatment, those with premature ovarian failure (POF) comprise an important subset as about 1% of all women will have this diagnosis [1]. Because POF is characterised by early loss of normal ovarian function before age 40, the unrecoverable loss of oocytes results in a bleak fertility prognosis unless oocyte donation with IVF is used.

Contemporary research has identified some genetic causes of POF, implicating a close association with Fragile × syndrome [2] and mutations in the inhibin alpha subunit gene [3]. Fragile × mental retardation-1 (FMR1) contains an unstable sequence of CGG trinucleotide repeats in its promoter region, with expansions of >200 trinucleotide repeats typically leading to abnormal methylation and silencing of FMR1 expression [4]. Therefore, in monozygotic (identical) twin sisters, any genomic anomaly linked to POF would be expected to negatively affect reproductive outcome in both siblings. The present report describes successful IVF for a POF patient where donated oocytes were obtained from her dizygotic (non-identical) twin sister.

## Methods and Results

A 33 year-old nulligravida presented with her husband for reproductive endocrinology evaluation. The couple had been attempting to achieve a pregnancy for 2 1/2 years. They had undergone two unsuccessful IVF cycles and a diagnosis of premature ovarian failure had been made after the second attempt. Neither partner smoked and both were in good general health. All screening laboratory tests were normal for husband and wife, however her serum FSH was elevated at 42 mIU/ml. Her karyotype was 46,XX. Intrauterine contours assessed via saline infusion sonogram were normal. The husband's semen analysis at our centre identified mild asthenozoospermia but was otherwise normal. Considerable patient counselling had already occurred after the two previous failed IVF treatments, and the couple understood that further cycles of IVF using native oocytes would probably be futile. However, the wife had a sister who had volunteered to serve as a known oocyte donor.

This sister (donor) was the dizygotic twin of the patient (recipient). She was married, had previously conceived without medical assistance and had a 10-month old child. The sister did not smoke, had a normal menstrual pattern and had discontinued breastfeeding four months before assessment. Her cycle day three FSH was 8.8 mIU/ml; all infectious disease and hormonal screening tests were normal. Additional counselling was undertaken for both couples (together and separately) in advance of the planned treatment and written informed consent was obtained from all parties [5]. Supplementary genetic testing for recipient and donor were declined.

After pituitary down-regulation with GnRH-agonist, the donor began a combined 225 IU/d FSH (Puregon^®^, Organon [Ireland] Ltd, Swords) plus 75 IU/d LH (Luveris^®^, Merck Serono Europe Ltd, London) superovulation regime. She received 10,000 IU hCG via subcutaneous injection on stimulation day eight with an uneventful transvaginal ultrasound-guided oocyte retrieval 36 h later. Ten oocytes were obtained and eight of these were metaphase II. Following ICSI with fresh sperm obtained from the recipient's husband, normal 2 *pn *fertilisation was noted in seven embryos.

Parallel to this, an appropriate endometrial response in the recipient was established by supplementary estrogen administration and verified by serial transvaginal ultrasound assessments. On day three, two fresh embryos were transferred to the recipient 11 mm inferior to the uterine fundus under ultrasound guidance; four embryos were cryopreserved the same day. Luteal support post-transfer was administered as previously described [6]. Pregnancy was confirmed 13 days post-transfer by positive hCG test, with a twin intrauterine gestation identified on follow-up obstetrical sonogram at seven weeks.

The patient had an unremarkable obstetrical course. Following a failed labour induction, she had a caesarean delivery at 39 weeks' gestation resulting in the birth of healthy male/male twins. Mother and babies were discharged from hospital five days post-partum in good condition; all continue to do well. Of note, an unassisted pregnancy was achieved by the sister-donor within six months of oocyte retrieval, which resulted in another singleton delivery of her own.

## Discussion

In the setting of the advanced reproductive technologies, the difficult diagnosis of premature ovarian failure is often followed by discussions about donor oocyte IVF. This treatment modality has become an important component of the comprehensive assisted fertility treatment programme, and has been available in Ireland for several years. Patient survey results from Irish patients have generally agreed with findings reported elsewhere, showing a strong preference for sister-donor over anonymous-donor oocyte approach in IVF [7]. Although successful sister-donor oocyte IVF has been available in Australia [8] and USA [9] for several years, this is the first description of IVF births resulting from this technique in Ireland. Indeed, because all prior births reported from sister oocyte donors involved a non-twin sibling as the gamete source, the current case appears to be the first in the medical literature validating a specific role for twin sister oocyte donation in IVF. Although IVF incorporating oocytes donated from a twin sister does not usually differ from ordinary (non-twin) sister oocyte donation, this case does extend the range of therapeutic options in POF to include a dizygotic twin as the oocyte source.

Although most POF cases probably result from *de novo *mutations [10], the genetics of this disorder are complex and have not yet been fully elucidated. Accordingly, any POF patient who contemplates sister oocyte donation should be advised about the possibility that any oocytes donated by a sister might carry a POF mutation [11,12]. Anonymous donor oocyte IVF is generally regarded as having a better reproductive outcome than IVF involving a related oocyte donor [13], and special ethical considerations exist with sibling gamete donation that do not apply in anonymous oocyte donation. Perhaps the most important of these is awareness of the resultant family dynamic following a birth of a child conceived from sister-donor oocyte IVF [14]. While counselling fully developed these issues, our patient nevertheless regarded the proven fertility of her (dizygotic) twin sister as sufficiently reassuring to proceed safely in the absence of genetic data. In this case satisfactory psychological assessments were completed, no absolute contraindication to known oocyte donation IVF was present, and ongoing counselling resources were available throughout the IVF sequence.

It must be acknowledged that few POF patients will have a sister willing and able to serve as a known oocyte donor for IVF. Accordingly, for most POF patients reproductive success will be achieved clinically from IVF using oocytes from an anonymous oocyte donor. But against the background of "routine" donor oocyte therapy for POF, the current report describes an IVF approach for the exceptional case where a dizygotic twin oocyte donor is available.

## Competing interests

The authors declare that they have no competing interests.

## Authors' contributions

ESS, ABO, DJW, US and APHW were consultants; ACB was medical student associated to the case. All authors read and approved the final manuscript.
